# A Study of Carbon Nanofibers and Active Carbon as Symmetric Supercapacitor in Aqueous Electrolyte: A Comparative Study

**DOI:** 10.1186/s11671-017-2415-z

**Published:** 2017-12-29

**Authors:** Allan Daraghmeh, Shahzad Hussain, Iyad Saadeddin, Llorenç Servera, Elena Xuriguera, Albert Cornet, Albert Cirera

**Affiliations:** 1 MIND, Engineering Department: Electronics, Universitat de Barcelona, Marti i Franquès 1, 08028 Barcelona, Spain; 2 Institute of Nanoscience and Nanotechnology (IN2UB), Universitat de Barcelona, Joan XXIII S/N, 08028 Barcelona, Spain; 3 Department of Physics, Al-Najah National University, P.O.Box 7, Nablus, West Bank Palestine; 4 EUSS, PasseigSant Joan Bosco,74, 08217 Barcelona, Spain

**Keywords:** Carbon nanofibers, Activated carbon, SEM, TEM, BET, Aqueous electrolyte, Supercapacitor

## Abstract

Symmetric supercapacitors are fabricated by carbon nanofibers (CNF) and activated carbon (AC) using similar proportions of 7 wt% polyvinylidene fluoride (PVDF) polymer binder in an aqueous electrolyte. In this study, a comparison of porous texture and electrochemical performances between CNFs and AC based supercapacitors was carried out. Electrodes were assembled in the cell without a current collector. The prepared electrodes of CNFs and AC present Brunauer-Emmett-Teller (BET) surface area of 83 and 1042 m^2^/g, respectively. The dominant pore structure for CNFs is mesoporous while for AC is micropore. The results showed that AC provided higher specific capacitance retention up to very fast scan rate of 500 mV/s. AC carbon had a specific capacitance of 334 F/g, and CNFs had 52 F/g at scan rate 5 mV/s in aqueous solution. Also, the results indicate the superior conductivity of CNFs in contrast to AC counterparts. The measured equivalent series resistance (ESR) showed a very small value for CNFs (0.28 Ω) in comparison to AC that has an ESR resistance of (3.72 Ω). Moreover, CNF delivered higher specific power (1860 W/kg) than that for AC (450 W/kg). On the other hand, AC gave higher specific energy (18.1 Wh/kg) than that for CNFs (2 Wh/kg).This indicates that the AC is good for energy applications. Whereas, CNF is good for power application. Indeed, the higher surface area will lead to higher specific capacitance and hence higher energy density for AC. For CNF, lower ESR is responsible for having higher power density.

Both CNF and AC supercapacitor exhibit an excellent charge-discharge stability up to 2500 cycles.

## Background

Supercapacitors or electrochemical capacitors have attracted much interest due to their high power density and long cycling capabilities. They have found potential applications in electric vehicles, portable devices, and power tools [1]. The electrical vehicles need high power at high current drain rate whereas memory backup systems require high energy density at low current drain rate. Consequently, the material should be chosen according to the desired applications [2]. The main components of a supercapacitor are the electrodes and the electrolyte. Since the charge storage takes place at the electrode/electrolyte interface, the surface area of the electrode and the electrolyte used will greatly influence the performance of the device. The electrode properties of like, material nature, electrode thickness, surface area, pore size distribution, and surface groups highly influence the performance of supercapacitor [3]. Carbon materials are widely used as electrodes due to their low cost, available diversity of morphologies, and chemical and thermal stability [4–7]. CNF nanoscale tubular morphology can offer a unique combination of low electrical resistivity and high porosity in a readily accessible structure [8]. An AC material is very attractive material for supercapacitors due to high porosity, low cost, abundance, high stability, and charge-discharge cycling [9]. The fabrication of electrodes (AC or CNFs) for supercapacitors requires the addition of binder—e.g., poly(tetrafluoroethylene) (PTFE), polyvynilidene chloride (PVDC), and polyvynilidene fluoride (PVDF)—in proportions that usually vary from 5 to 10 wt. % in order to maintain the integrity of electrodes [10, 11]. However, binder blocks the part of porosity of carbon and additionally causes an increase in electrical resistivity [11–13].

The capacitance of supercapacitor is highly linked to the electrode material and the electrolyte. The electrolyte compatibility with the electrode material also plays a crucial role in the development of supercapacitor because the electric double layer is built at the electrode/electrolyte interface. The voltage of a supercapacitor depends on the stability potential window of the electrolyte. The aqueous electrolytes usually provide potential until 1.0 V and organic electrolyte until 2.7 V [14]. Aqueous electrolytes are environmentally friendly, whereas organic electrolytes are not good environmentally. Aqueous electrolytes are mostly composed of small anions and simple hydrated cation (angstrom level). These ions can easily penetrate to the micropores, mesopores, and macropores of the material under the applied electric field. The electric double layer (EDL) built at the electrode/electrolyte interfacial region can be treated as a capacitor with an electric double layer capacitor (EDLC), which can be expressed as *C* = *ϵA*/*d*. Where *ϵ* is electrolyte dielectric constant, *A* is the surface area accessible to ions, and *d* is the distance from ions to the pore surface of carbon electrode on the order of an angstrom. According to the above equation, two approaches can be taken to enhance the charge storage of EDLC effectively: increasing the SSA and reducing the distance between ions and the carbon surface by the development [15].

In this work, the aim is to provide a comparative analysis of symmetric supercapacitor based on AC and CNFs by using a similar amount of binder PVDF 7 wt% for both materials.

## Methods/Experimental

### Preparation of AC and CNF Electrodes

Symmetric supercapacitor based on AC and CNFs was prepared for comparison. AC reference Carbopal CCP80 from Donau Carbon is supplied by QuimicsDalmau. CNFs have a helicoidally graphitic stacked cup structure, there is a presence of Ni (6%), the diameter is 20–80 nm, length (MEB) > 30um, and electric resistivity of 10^−2^ Ω cm.

PVDF was used as a binder. In order to compare electrode preparation for supercapacitor analysis for both materials (AC, CNFs) was achieved in a similar way by following the below steps.

Step 1: Milling of (AC or CNFs) in a zirconia planetary ball mill (Pulverisette 7 from Fritch) employing a frequency 500 rpm for 30 min. Step 2: Mixing of AC or CNFs 93 wt% with 7 wt% PVDF polymer by using 15 ml acetone in an agate mortar. Step 3: The slurry was then mixed using a mechanical stirrer for 60 min followed by an ultrasonic for 30 min. Step 4: The slurry of the mixture was dried in an oven for 60 min at 70 °C. Step 5: In the last step, the dried slurry was used to prepare the electrodes, in a way, using a hydraulic press with a die set (10 mm) at 10 tones force. The calculated mass of the prepared electrode discs based on CNFs and AC were 0.018 and 0.02 g, respectively.

### Surface Characterization

The porous texture and specific surface area and pore size distribution of CNF and AC electrodes were obtained by physical adsorption of gasesN_2_ at 77 K using Micromeritics TriStar 3000 V6.04 A .All samples were outgassed at 100 °C for 4 h prior to the adsorption measurements. The specific surface area (*S*_BET_, m^2^/g) was determined by multipoint Brunauer-Emmett-Teller (BET) method in the region of the isotherm, which is limited by the range of relative pressure *P*/*P*_0_ = 0.02–0.2. The total volume of pores (*V*_total_, cm^3^/g) was calculated by the number of adsorbed nitrogen at *P*/*P*_0_ ≈ 0.9932. The volume of micropores and the values of surface areas of micro (*S*_micro_, m^2^/g) were investigated by using t-plot (Harkins and Jura) method; the pore size distribution for CNF sample is calculated from adsorption isotherms by the Barrett-Joyner-Halenda (BJH) method; and MP method is used to calculate the pore size distribution for AC.

### Morphological Characterization

The AC and CNF samples were examined using scanning electron microscopy (SEM). TEM analyses were performed on a Philips Tecnai G2 F20 system operated at 300 kV. The samples were suspended in ethanol and dispersed ultrasonically for 15 min. A drop of the suspension was deposited on a copper grid coated with carbon.

### Electrochemical Characterization

The electrochemical performance comparison of AC and CNFs as symmetric capacitors was studied in two electrode Swagelok cells and using a Gamry 600 potentiostat using 6-M KOH solution as an electrolyte. The specific capacitance of electrode materials was investigated by cyclic voltammetry (CV), galvanostatic charging/discharging (GCD), and electrochemical impedance spectroscopy (EIS).

## Results and Discussion

### Morphological Characterization

**Fig. 1:**
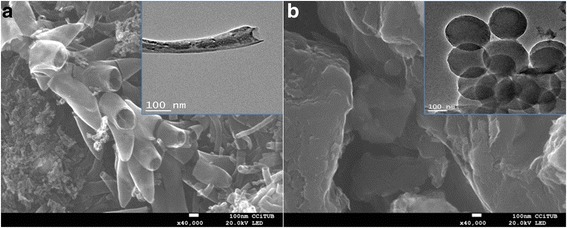
SEM images and TEM images (inset) for **a** CNFs and **b** AC

The surface morphology of prepared electrodes was investigated by SEM Fig. 1 and TEM Fig. 1 (inset). It can be seen clearly that PVDF binder effectively bonds the CNFs Fig. 1a and AC Fig. 1b. The different structures for both electrodes of CNFs and AC are visible. The typical CNF structure, cylindrical shape, and crystals structure inset Fig. 1a. TEM image of AC demonstrates interconnected spheres with homogeneous size and smoother surface inset Fig. 1b.

### Pore Texture of CNFs and AC

**Fig. 2:**
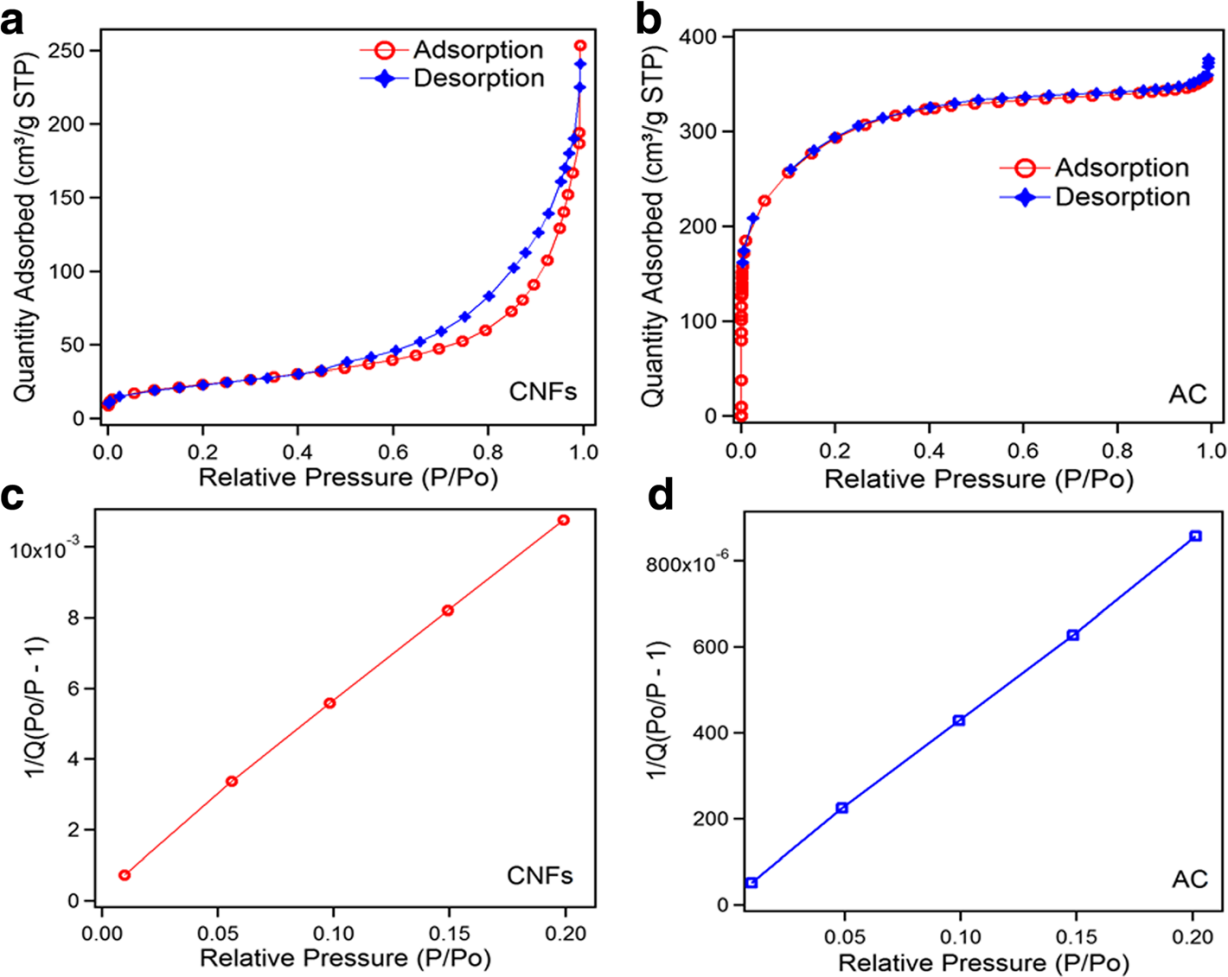
Nitrogen adsorption/desorption isotherms, **a** CNFs and **b** AC. BET surface area **c** CNFS and **d** AC

The N_2_ adsorption/desorption isotherm of CNFs and AC is shown in Fig. 2. Pore volume and pore size distributions were calculated by BJH method, t-plot method, and MP method. Only MP method analysis can reveal the fine difference of micropore size distribution of the sample [16]. The pore size distribution of the materials is classified into three groups: micropores (< 2 nm), mesopores (2–50 nm), and macropores (> 50 nm) [17]. The isotherm of CNFs presents a small hysteresis loop from higher to middle pressure range, which indicates CNFs contain mesoporous structure. Due to that only BJH method is used for pore size detection since MP method cannot detect meso- and macroporosity. According to IUPAC classification, the isotherm of CNFs can be classified as type II isotherm. The pore distribution of CNFs is as follows: 59% mesopores (2–50 nm), 17.9% micropores (0.5–2 nm), and 23% macropore (> 50 nm). The details are presented in Table 1.

**Table 1:** Physicochemical parameters of CNFs and AC

Pore size distribution							*P*%					
Sample	BET^a^, m^2^/g	*V*_t_^b^, cm^3^/g	*V*_0.5-2_^c^, cm^3^/g ,t-plot	*V*0._2–0.5_^d^, cm^3^/g, MP	*V*_2-50_^e^, cm^3^/g, BJH	*V*_> 50_^f^	0.5–2^g^	0.2–0.5^g^	2–50^g^	> 50^g^	APS, nm	Ext, m^2^/g
CNFs	83	0.39	0.07	–	0.23	0.09	17.9	–	59	23	4–7.5	157.4
AC	1042	0.582	0.19	0.32	0.072	–	33	55	12	–	0.47	21

^a^BET surface area

^b^Single-point volume adsorption total volume of pores at *P*/*P*_0_ = 0.9932

^c^Supermicro volume from 0.5 and 2 nm t-plot, y-intercept

^d^Ultramicro volume from MP method

^e^Meso volume from BJH method

^f^Volume greater than 50 nm by BJH method

^g^The micro and meso percentage calculated by *V*_MICR_/*V*_TOTAL_ × 100%, *V*_MESO_/*V*_TOTAL_ × 100%, APS (average pore size), EXT (external area) from the slope of t-plot

AC adsorption/desorption isotherm presents that most of the adsorption quantity takes place at very low relative pressure (*P*/*P*_0_ ≤ 0.02) and a plateau from low to high relative pressure (0.6–0.8). The total pore volume is 0.582 cm^3^/g at relative pressure (*P*/*P*_0_ = 0.9932). Figure 2b presents that the curvature of isotherm from 0 to 0.4 relative pressure presents pore volume for less than 50 nm pores (micro + meso), and this pore volume is equal to 0.534 cm^3^/g which is the indication of highly microporous structure. The system of AC sample isotherm is classified as type I isotherm. The pore distribution of AC is as follows: supermicropores (0.5–2 nm) occupied 33 %, ultramicro (0.2–0.5 nm) occupied 55%, and mesopores occupied 12%. The MP method was used for AC pore size detection because BJH method cannot detect AC microporosity. The details are presented in Table 1.

The specific surface area (BET) was determined by multiple point Brunauer-Emmett-Teller (BET) method in the region of the isotherm, which is limited by the range of relative pressure *P*/*P*_0_ = 0.02–0.2 as seen in Fig. 2c, d. The total volume of pores (V_total_, cm^2^/g) was calculated by the number of adsorbed nitrogen at *P*/*P*_0_ ≈ 0.9932. The adsorption volume shows that BET surface areas for CNFs and AC are 83 and 1042 m^2^/g, respectively.

**Fig. 3:**
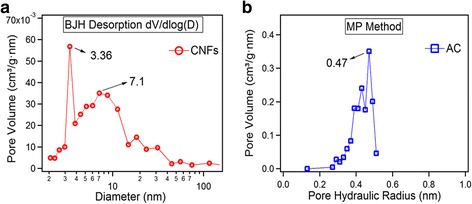
Pore size distribution. **a** CNFs by BJH method. **b** AC by MP method

The pore size distribution analyses are presented in Fig. 3a, b obtained via MP method for AC and using Barrett-Joiner-Halenda (BJH) method for CNFs. CNFs contain two types of dominant pores centered in the ranges of 3.36 and 7.1 nm, while AC is mainly comprised of pores of 0.47 nm. Microspores are beneficial for a charge accumulation in aqueous electrolytes [18, 19]. It can be seen that for CNFs, most dominant pores are mesopores while for AC ultra-micro pores.

### Electrochemical Behavior of CNFs and AC

The main accepted approaches to evaluate the capacitance of supercapacitor are cyclic voltammetry, galvanostatic charge/discharge, and impedance spectroscopy. The working principle of each technique varies from one to another. The electrochemical behavior of AC and CNFs was first characterized by cyclic voltammetry in the range of 0 to 1 V. CV is the most convenient method to characterize the capacitive behavior of electrode materials. The specific capacitance per unit mass for one electrode was calculated using Eqs. (1, 2).

1 $$ {C}_{\mathrm{s}}=4\times C/m $$

2 $$ C=\frac{q_{\mathrm{a}}+\left|{q}_{\mathrm{c}}\right|}{\Delta  V} $$

Where *C*_s_ is the specific capacitance in F/g, *C* is the measured capacitance for the two-electrode cell by Eq. 2, and *m* is the total mass of the active material in both electrodes [20].

**Fig. 4:**
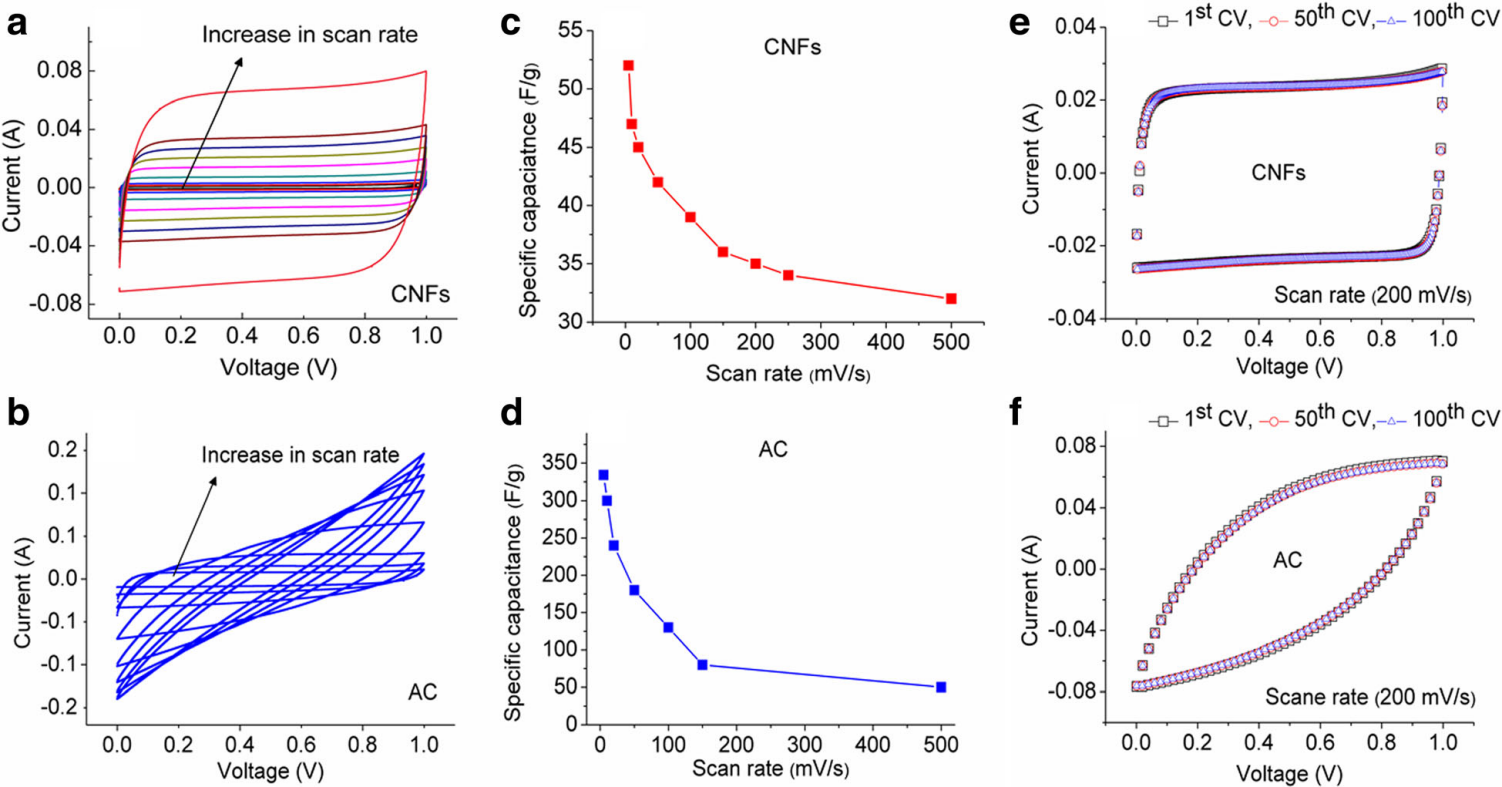
**a**, **b** CVs of CNFs and AC, respectively, at 5, 10, 20, 50, 100, 150, and 500 mV/s scan rates; **c**, **d** specific capacitance comparison at different scan rates; **e**, **f** CVs of CNFs and AC, respectively, from 1st to 100th cycle

Figure 4a, b shows the CVs of CNFs and AC, respectively, from 5 to 500 mV/s scan rates. CVs of CNFs in a wide range of scan rates are near box-like shape without any hump, or deviation indicates clear double-layer characteristics and high reversibility. The CVs for AC show much higher current than the CNFs. At low scan rates, the CV shape is rectangular, presenting that the electrode response in charging and discharging is highly reversible. However, at higher scan rates, CV deviates from a rectangular shape. There could be several possible reasons related to this deviation, (1) due to low electrical conductivity of porous structure of AC of inner pores that are not accessible to ions and (2) nonzero time constant and elevated transient current, resulting in a longer capacitor charging time and a collapse of the rectangular shape [21, 22].

Figure 4c, d presents the specific capacitance comparison of CNFs and AC from 5 to 500 mV/s scan rates. As it can be seen in Fig. 4c, CNFs show highest specific capacitance 52 F/g at scan rate of 5 mV/s. The specific capacitance decreased to 32 F/g at 500 mV/s scan rate. These results indicate the moderate decrease in specific capacitance, and at even higher scan rates, most of the surface area and pores of CNFs are accessible to the ions. The capacitance of AC decreases from 334 to 50 F/g for 5 to 500 mV/s Fig. 4d. Very high specific capacitance at low scan rate is due to ions have enough time to go deep inside the microporous (less than 2 nm) structure of AC. It can be presumed that at higher scan rates, mostly the larger pores mesopores (2–50 nm) are contributing to capacitance. This is mainly due to the difference in diffusion rate of electrolyte in the pores of different size and also due to network connection between large and small pores [23]. As discussed by A.G. Pandolofo et al., the measured surface area is contributed by the all the open pores, but all pores are not accessibly electrochemically [8].

The higher specific capacitance of AC in comparison to CNFs could be due to the higher surface area, which results in the increase in the accessible areas for electrolyte ions for charge storage within relatively small pores.

The CV curves of the CNFs and AC (Fig. 4e, f) indicate stable capacitance behavior measured until 100th cycle at a scan rate of 200 mV/s. The 100th CV cycle for both samples retain the shape as it was for 1st cycle suggests excellent stability and reversible electrode processes.

**Fig. 5:**
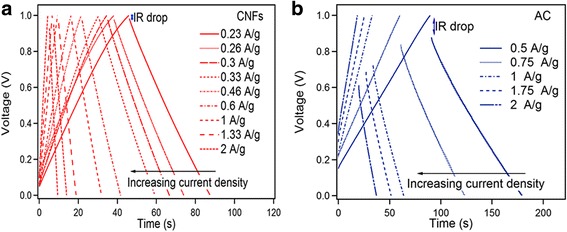
**a** GCD curves at different current densities of CNFs. **b** GCD curves at different current densities of AC

The supercapacitive performance of CNFs and AC was further compared by GCD as shown in Fig. 5a, b. The discharge capacitance (*C*) is estimated from the slope (*dV/dt*) of the linear portion of the discharge curve using Eq. 3.

3 $$ {C}_{\mathrm{s}}=\left(\frac{2I}{\left( dV/ dt\right).m}\right) $$

Where *C*_s_ is the specific capacitance in F/g, *∆V* is the voltage difference during the discharge curve in *V*, *I* is the current in *A*, and *∆t* is the discharge time in *s*.

**Fig. 6:**
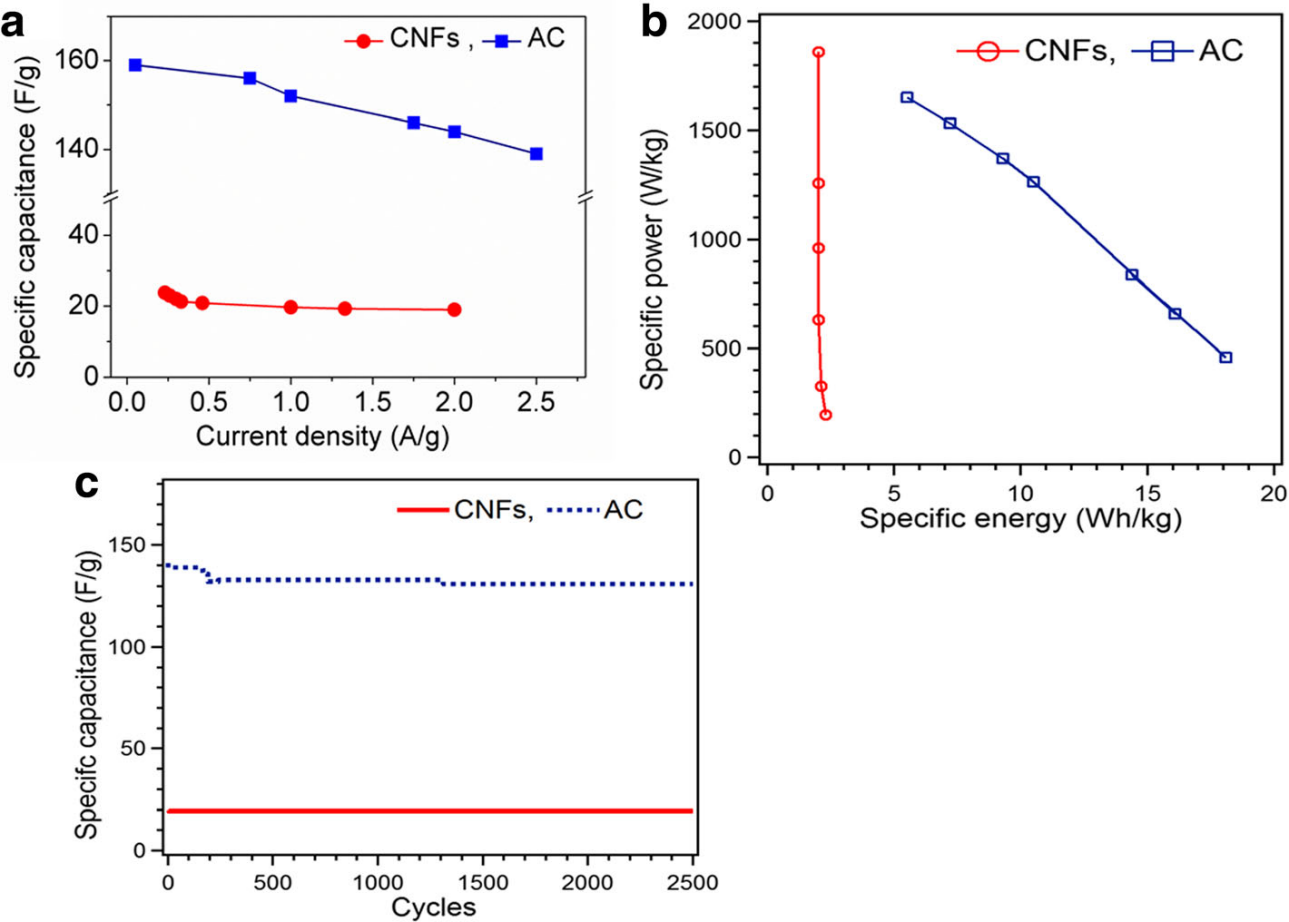
**a** Specific capacitance comparison from discharge curve of GCD. **b** Ragone plot of specific power against specific energy for CNFs and AC. **c** Cycling stability of CNFs and AC

It can be seen that the charging and discharging processes are nearly symmetric, indicating excellent electrochemical reversibility of the electrodes. The discharging curves of CNFs show a small *IR* drop implying a small equivalent series resistance, which is essential to power characteristic of supercapacitors. The lower *IR* drop of CNFs to the AC is due to the high conductivity of CNFs. The large *IR* drop for AC means higher equivalent series resistance (ESR). The calculated specific capacitance for CNFs 23.8 F/g at 0.23 A/g decreases to 19 F/g at 2 A/g. The specific capacitance of AC decreases from 159 F/g at current density 0.5 A/g to 139  F/g at 2.5 A/g Fig. 6a. The specific capacitance of both symmetric capacitors of CNFs and AC decreases with the increase of current density, which is very common for supercapacitors and is mainly caused by the diffusion limitation of electrolyte ions in the microspores of the electrode.

The specific power, *P*, and specific energy, E, delivered upon discharge were estimated by Eqs. (4) and (5).

4 $$ P=\raisebox{1ex}{$ Vi$}\!\left/ \!\raisebox{-1ex}{$m$}\right. $$

5 $$ E=\raisebox{1ex}{$ Vit$}\!\left/ \!\raisebox{-1ex}{$m$}\right. $$

Where *V* is the voltage excluding *IR* drop, *i* is discharge current, and *t* is the time [24].

As can be seen in the Ragone plot Fig. 6b, the CNF electrode shows a maximum specific energy of 2.3 Wh/kg at a specific power of 197 W/kg and a maximum power density of 1860 W/kg at a specific energy of 2 Wh/kg indicating its good power characteristics. These results show an increase of specific power; the specific energy only decreases a little, which is a signature of excellent electrochemical properties of high energy density and power output, therefore very promising for application in the scenarios where high power output as well as high energy capacity is required [25]. For AC with the increase in specific power from 459 to1650 W/kg, the specific energy decreased from 18.1 to 5.5 Wh/kg.

The cycling stability is also a vital factor for practical applications. Galvanostatic charge-discharge cycling measurement was performed at a constant current density 2 A/g for AC and for CNFs up to 2500 cycles Fig. 6c. The behavior of AC during cycling stability shows a small decrease in capacitance from 141 to 131 F/g in 2500 cycles. This result expresses that the capacitance fading is due to the irreversible reactions at the beginning of the cycling [10]. The CNF cycling measurements reveal excellent capacitance retention 19 F/g in 2500 cycles.

**Fig. 7:**
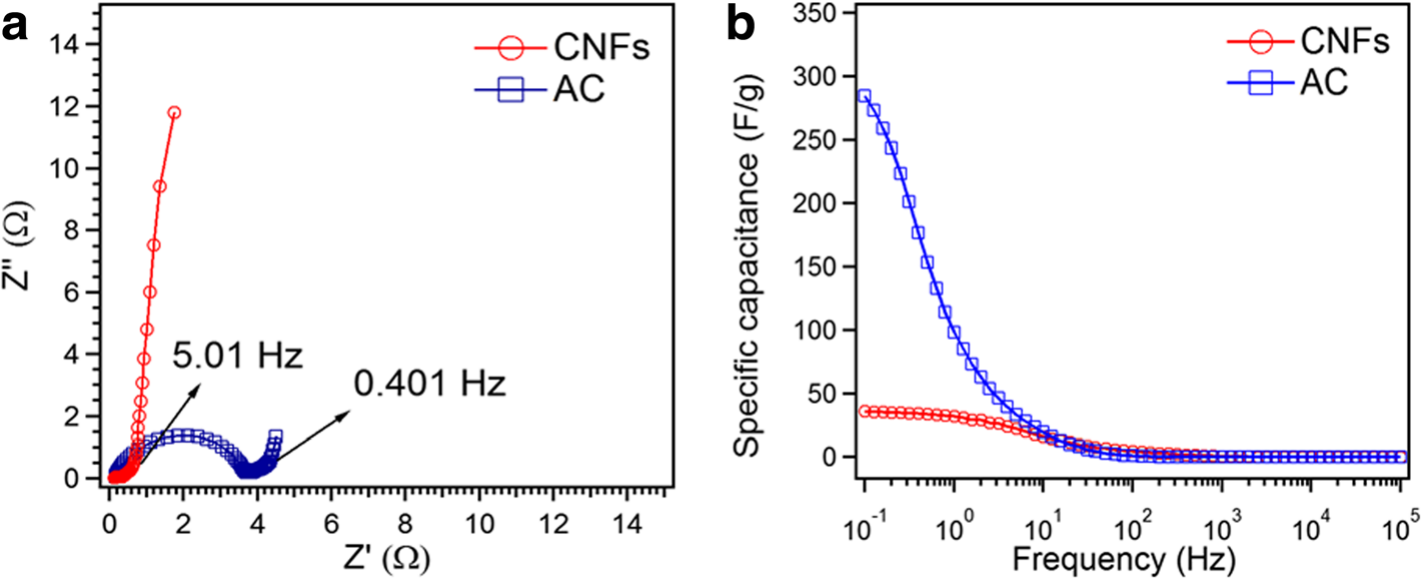
**a** Nyquist plot of CNFs and AC. **b** Csp comparison calculated from EIS.

The supercapacitor was further analyzed by electrochemical impedance spectroscopy (EIS). It depicts the Nyquist plot at in the frequency range of 10 kHz–0.1 Hz for CNFs and AC (Fig. 7a). The Nyquist plots consist of (1) a high-frequency intercept on the real Z’-axis, (2) a semicircle in the high-to-medium-frequency region, and (3) a straight line at the very low-frequency region [26]. In the high-frequency region, the intercepts with Z’ of CNFs and AC are 0.11 and 0.16 Ω, respectively. This value is considered as the total electrical resistance of the electrode material, electrolyte, and electrical contacts [27]. The semicircle from high to medium frequency corresponds to parallel combination of charge transfer resistance (*R*_ct_) and double layer capacitance [28]. It can be seen that semicircle (*R*_ct_) is higher for AC (3.56 Ω) than that for CNFs (0.17 Ω). The calculated ESRs were 0.28 and 3.72 Ω for CNFs and AC, respectively. The very small value of ESR for CNFs, relative to AC, indicates the facile electron and ion transport/diffusion in the CNF electrodes. This indicates that CNF electrode has much higher conductivity than AC electrode. As also observed in Fig. 7a, CNFs showed a higher slope of the straight line, in the low frequency range, than the AC. This means that CNFs exhibit a higher capacitive behavior than AC.

The specific capacitance, *C*_s_, of the CNF and AC supercapacitor was also calculated from the impedance analysis employing the imaginary component of the impedance by following equation [29].

6 $$ {C}_{\mathrm{s}}=4\left(-\left(1/2\pi f{z}^{\prime \prime }m\right)\right) $$

Where *f* is frequency in Hz, *z*” is the imaginary component of impedance, and *m* is the mass of CNFs or AC calculated for one electrode. Figure 7b shows the higher change in the specific capacitance of CNFs and AC below the frequency of 10 Hz. The obtained specific capacitance for CNFs (36 F/g) and AC (284 F/g) at the frequency of 0.1 Hz is pretty much comparable to the capacitance calculated by CV. Indeed, higher specific capacitance for AC is due to higher surface area available for charge accumulating at the solid liquid interface.

Time constant *τ* is the property of the supercapacitor which reflects the response of the device. A small value of *τ* gives indication of better response. The time constant *τ* was calculated using the following equation:

7 $$ 2\tau =\frac{E_{\mathrm{D}}}{P_{\mathrm{D}}} $$

Where *E*_D_ is the energy density and *P*_D_ is the power density. *E*_D _and *P*_D_were calculated using the following equations:

8 $$ {E}_{\mathrm{D}}=0.5C{V}^2/m $$

9 $$ {P}_{\mathrm{D}}={V}^2/4\left(\mathrm{ESR}\right)m $$

Where *V* is the voltage window during charge discharge curve, *C* is the capacitance from the charge-discharge and ESR calculated from impedance spectroscopy, and *m* is the mass of electrode. The calculated time constant *τ* for AC was 3.1 s and for CNFs was 0.08 s at current density 2 A/g, indicating a better capacitive response for CNFs.

**Fig. 8:**
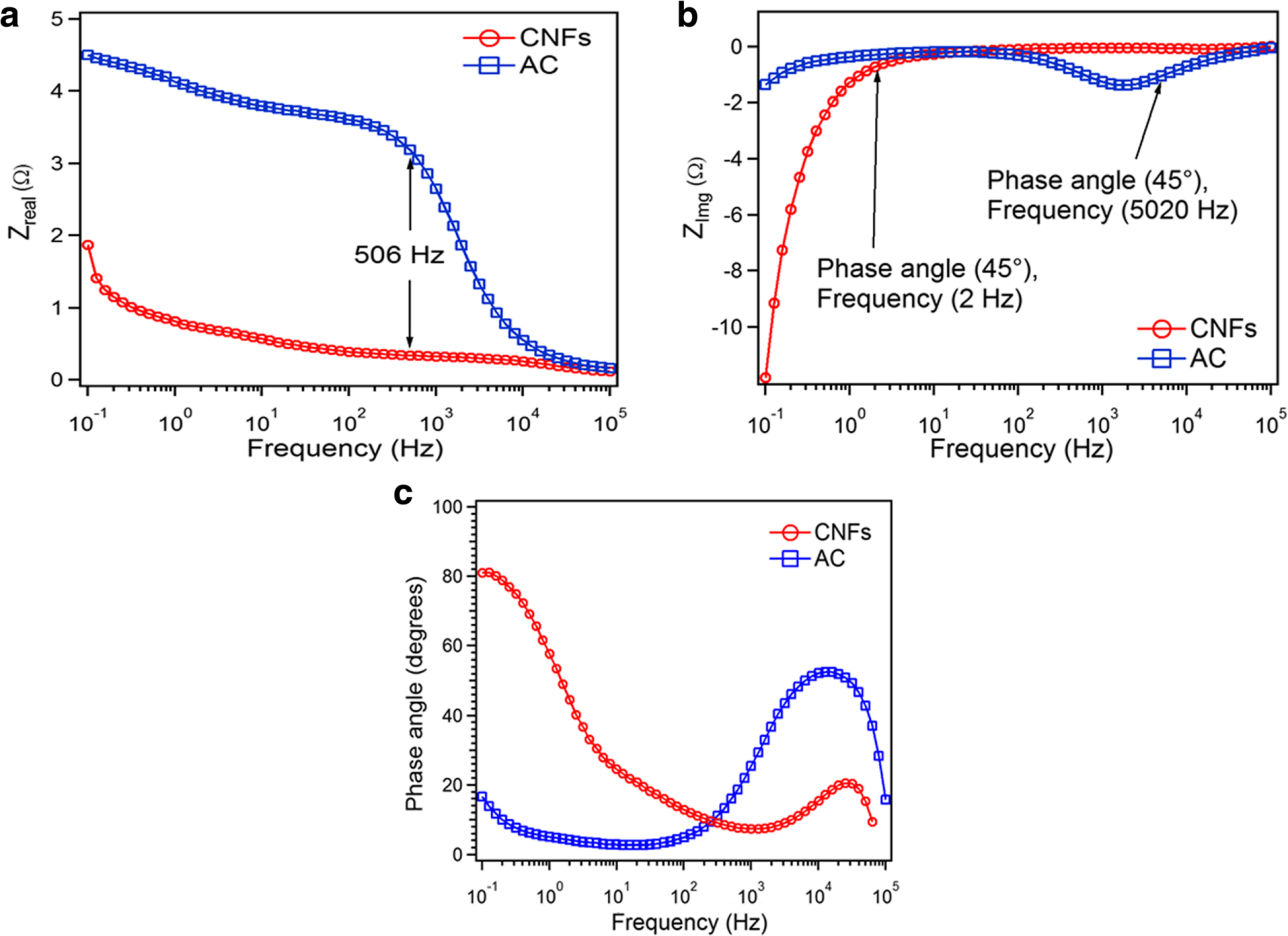
**a** The real and **b** imaginary parts are plotted as a function of log of frequency and **c** phase shift as function of frequency for AC and CNFs

The relationship between *Z*_real_ and frequency gives us information about the electrolyte and charge transfer resistance in electrolyte Fig. 8a. The resistance behavior of electrode is greatly influenced by the nature of carbon electrode. For both CNFs and AC at high frequency 100 KHz, ESR is at their lowest values in the order of about 0.1 Ω, which presents the electrolyte resistance *R*s. With lowering down the frequency, until 506 Hz, there is a sharp increase in the resistance of AC relative to CNFs. At the lowest observed frequency (0.1 Hz), ESR was observed to have a value of 1.87 and 4.5 Ω for AC and CNFs, respectively. The increase in ESR with decreasing frequency could be due to the difficulty of penetration of the electric signal into the deeper pores (filled with electrolyte) and/or in the smaller particles [30]. This variation can be justified by the fact that as the frequency decreases, ions can easily reach the deeper zones of the activated carbon pores, and consequently, their longer displacement within the electrolyte results in higher electrolyte resistance [31].

The relationship between *Z*_Img_ and frequency gives us information about relaxation time (*τ*_0_) at the boundary region, where supercapacitor transfers from resistor to capacitor Fig. 8b. The time constant *τ*_0_ that corresponds to the phase angle 45° represents the transition of electrochemical capacitor from a purely resistive to purely capacitive behavior. It is well known that higher power delivery corresponds to lower *τ*_0_ values. For frequency, f > 1/*τ*_0_, it acts as a pure resistor, and for f < 1/*τ*_0_, it behaves as a pure capacitor. The 45° phase angle was found for AC at frequency 5020 Hz and for CNFs at 1.99 Hz. This shows that relaxation time for AC is much less than that for CNFs. Hence, translation resistive behavior to capacitive behavior for AC is much faster than CNFs.

Figure 8c represents the variation of phase angle as a function of frequency, which is known as Bode plot. The phase angles are found to be − 20° and − 88° at low frequency 0.1 Hz in the AC and CNF systems, respectively (Fig. 8c). In general, phase angle approach to − 90° confirms better capacitive performance and rapid charge-discharge process. The relaxation time constant, *τ*0, defines the time required to deliver the stored charge effectively as seen in Fig. 8b [32].

### Effect of Carbon Structure and Porous Texture on EDLC Performance

From electrochemical characterization, it is evident that supercapacitor based on AC electrodes gives higher specific capacitance than CNFs in 6-M KOH electrolyte. According to the equation, ∁ =  ∈ *A*/*d*, distance (*d*) is very small when the electrode contains micropores. The higher capacitance of AC is due to some important properties, those are higher BET surface area and existence of higher 88% of ultramicropores and micropores. Whereas, the CNF samples have low BET surface area and 17.9% of micropores. Another important factor that influence the capacitance is related to the following equation, *τ* = *L* ^2^/*D*. Where *L* refers to the ion transport length, and *D* refers to the ion transport coefficient. According to this equation, the ions enter fast inside the micropores, but as the size of pores increase, the external area also increase. Due to this fact, the ions accumulate outside of the pores, hence result in the decrease in the capacitance. According to E. Raymundo-Pinero et al., in aqueous solution, the double layer formation is much favorable when the pore size is around 0.7 nm [19]. Our results reveal that pore size of AC (0.47 nm) is in the optimal range to build the double layer, hence presents higher specific capacitance over CNFs.

## Conclusions

CNF and AC electrodes have been prepared in a similar technique and compared as symmetric supercapacitor using aqueous solution. Pore size distribution, surface area of the electrode, and total electrode resistance were found to play a crucial role in determining the supercapacitor performance. BET results reveal that AC has a high number of micropores and ultramicropore structure that gives a surface area of 1042 m^2^/g, whereas CNF electrode contains dominant mesosporous structure and surface area 83 m^2^/g. Due to that, AC material delivers a specific capacitance (334 F/g) much higher than CNFs (52 F/g). Indeed, a higher specific capacitance for AC gave a higher specific energy (18.1 Wh/kg) than that for CNFs (2 Wh/kg). On the other hand, CNFs reveal lower ESR (0.28 Ω) than AC (3.72 Ω). The obtained specific powers depending on ESR value were 1860 and 450 W/kg for CNFs and AC, respectively. Hence, AC is considered to be suitable for energy applications. Whereas, CNF is a better candidate for power applications.
